# Retention of Zn, Fe and phytic acid in parboiled biofortified and non-biofortified rice

**DOI:** 10.1016/j.fochx.2020.100105

**Published:** 2020-09-29

**Authors:** Víctor Taleon, Sonia Gallego, Juan Camilo Orozco, Cecile Grenier

**Affiliations:** a HarvestPlus, c/o International Food Policy Research Institute (IFPRI), 1201 Eye Street, NW, Washington, DC 20005, USA; b HarvestPlus, The Alliance of Bioversity International and the International Center for Tropical Agriculture (CIAT), Cali, Colombia; c CIRAD, UMR AGAP, F-34398 Montpellier, France SupAgro, Montpellier, France; d AGAP, Univ Montpellier, CIRAD, INRAE, Montpellier, France

**Keywords:** Parboiling, Zn retention, Zn rice, Biofortification, Degree of milling

## Abstract

Biofortified rice with high Zn concentration could reduce Zn deficiency in South Asia. This population frequently parboils rice. True retention (TR) of Zn, Fe and phytic acid after parboiling and milling was evaluated in biofortified and non-biofortified rice. TR in milled non-parboiled rice was 63.8–89.6% for Zn, 21.1–44.5% for Fe and 16.4–40.3% for phytic acid, whereas in milled parboiled rice TR was 49.8–72.2% for Zn, 23.4–36.7% for Fe and 22.0–33.3% for phytic acid. Milled parboiled rice resulted in lower Zn TR compared to milled non-parboiled. These results suggest that Zn moves from the inner endosperm towards the outer layers during parboiling, regardless of initial Zn concentration, consequently, once milled, the potential impact of Zn intake on Zn deficiency from parboiled rice is less than non-parboiled rice. Despite Zn losses during processing, biofortified rice could provide over 50% of the Zn EAR for children.

## Highlights

• Zinc in rice endosperm moved to the outer layers during parboiling.
• Zinc retention in parboiled rice was lower than non-parboiled rice after milling.
• Biofortified rice had higher zinc retention than non-biofortified.
• Biofortified rice could provide more than half of the zinc EAR% for children.

## 1 Introduction

In several countries where rice is the main staple cereal food, Zn intake is below the estimated average requirement (EAR), in part because common commercial rice varieties contain low levels of Zn (11**–**16 µg.g^−1^) after milling (Mayer et al., 2008). An increase of 12 µg.g^−1^ of Zn in milled rice could provide at least 25% of the Zn EAR for preschool children. Therefore, biofortified rice varieties with up to 28 µg.g^−1^ of Zn in the milled grain are being developed through conventional plant breeding to improve the Zn intake of deficient populations in Asia and Latin America (Trijatmiko et al., 2016, Andersson et al., 2017).

While milled (white) rice is the most common form of rice consumed globally, in South Asia it is also common to consume milled parboiled rice (Oli et al., 2016, Bairagi et al., 2020). To parboil rice, paddy is soaked, heat treated and dried before dehulling to produce parboiled brown rice and then milled to the desired degree of whiteness (Oli et al., 2014).

Milled parboiled rice contains high levels of B vitamins compared to milled non-parboiled rice. For this reason, it is generally believed to be more nutritious (Oli et al., 2014, Runge et al., 2019). Also, parboiling can be used to fortify rice with minerals and vitamins by adding them to the soaking water (Patindol et al., 2017, Jannasch and Wang, 2020). However, the concentrations of Zn and Fe, two nutrients of significant public health importance in South Asia, in parboiled (non-fortified) rice may be lower than in non-parboiled rice, depending on the processing methods used. Mayer et al. (2008) evaluated the impact of milling on parboiled rice from Bangladesh and reported 24**–**39% lower Zn concentration in milled compared to unmilled (brown) parboiled rice. Biswas et al. (2018) reported that Zn concentration in milled parboiled rice was 8**–**20% lower compared to brown parboiled rice but it was 13**–**28% higher than milled non-parboiled rice. In contrast, Denardin et al. (2004) reported 44% lower Zn concentration in milled parboiled rice compared to milled non-parboiled rice, without reporting the degree of milling (DOM). Furthermore, two surveys in Brazilian markets showed that Zn concentration in milled parboiled rice was lower compared to milled non-parboiled rice (Da Silva et al., 2013, Runge et al., 2019). The Fe concentration found by Da Silva et al. (2013) was twice as high in milled parboiled rice compared to milled non-parboiled rice, whereas Runge et al. (2019) did not find differences between these two types of rice (DOM unreported).

Rice also contains phytic acid (PA) which is a chelator of divalent minerals such as Zn and Fe and limit their absorption (Kumar et al., 2017). Given the impact of PA on Zn and Fe absorption, the ratio between PA and Zn and Fe is commonly used to better understand how well the mineral could be absorbed in the body (Hambidge et al., 2008, Al Hasan et al., 2016). Liang et al. (2008) reported that most PA in non-parboiled rice is concentrated in the outer layers of the grain and is removed during milling, however, it is unknown how parboiling affects PA concentration of milled rice.

Zn, Fe and PA concentrations are affected by parboiling and milling but the magnitude of the effect on biofortified rice versus non-biofortified rice, which have contrasting zinc concentration, has not been evaluated under controlled parboiling and milling conditions. Therefore, this study evaluated the retention of Zn, Fe and PA in parboiled high Zn rice with and without milling, applying methods of processing commonly used by South Asian communities. This information is a key determinant to understanding the potential impact on Zn intake from consumption of traditionally prepared biofortified rice.

## 2 Materials and methods

### 2.1 Rice samples

Three biofortified (BF) and two non-biofortified (NBF) rice entries were evaluated. BF14AR035 (BF2) and BF14AR050 (BF3), two elite breeding lines from the International Center for Tropical Agriculture (CIAT), and variety BRRI dhan64 (BF1) released in Bangladesh, were the BF entries used in this study. The NBF entries were the control variety IR64 (NBF1) and a rice variety locally grown in Colombia, FEDEARROZ68 (NBF2). The rice was cultivated in two experimental stations with contrasting conditions in Colombia: Palmira-Valle del Cauca, under irrigated conditions at the CIAT-HQ, and Santa Rosa, located in Villavicencio-Meta, under favorable rainfed conditions at the FEDEARROZ Research Center (Table 1). The biofortified entries were developed using conventional breeding methods. Paddy rice was harvested at crop maturity in Palmira (March 2017) and in Santa Rosa (August 2017). Moisture of paddy during harvest was 24**–**28%. Panicles (before threshing) and paddy (after threshing, before parboiling) were sundried in a clean plastic surface, in a clean open space to minimize dust in the surroundings. Paddy was sundried to reach 10**–**12%. After drying, the paddy was sieved to remove dust and other particles. Grain weight, dimensions, and amylose content were measured. Fat content and whiteness (Kett degrees) of milled rice was also measured. The whiteness was measured with a commercial whiteness meter (Rice Milling Meter, Model MM1D, Satake Co., Japan). Raw paddy (RAW) subsamples (400 g) were taken before soaking for grain characterization and analysis.

**Table 1:** Zn, Fe, PA, PA:Zn and PA:Fe, thousand kernel weight (TKW) and amylose content in brown non-parboiled rice (NPBDOM0) of three biofortified and two non-biofortified entries evaluated at two locations in Colombia¥.

Location	Grain entry	Grain source code	Zn (µg.g^−1^)	Fe (µg.g^−1^)	PA (mg.g^−1^)	PA:Zn ratio	PA:Fe ratio	TKW (g)	Amylose (%)
Palmira	BRRI Dhan64	BF1P	17.0 ± 0.4^e^	10.5 ± 0.1^c^	10.2 ± 0.2^bc^	59.2 ± 0.9^b^	82.2 ± 2.2^abc^	20.8 ± 0.5 ^cd^	25.5 ± 1.4^bc^
	BF14AR035	BF2P	20.1 ± 0.9^d^	11.2 ± 0.5^bc^	9.7 ± 0.1 ^cd^	47.6 ± 1.8^c^	73.3 ± 2.6^bc^	23.1 ± 0.3^b^	28.4 ± 0.8^a^
	BF14AR050	BF3P	20.5 ± 1.6^d^	13.2 ± 0.6^a^	11.6 ± 0.8^a^	56.5 ± 7.9^bc^	74.3 ± 5.9^bc^	24.7 ± 0.1^a^	22.0 ± 1.4^d^
	IR64	NBF1P	13.4 ± 0.6^f^	9.2 ± 0.5^def^	10.0 ± 0.2^bcd^	73.4 ± 2.9^a^	91.8 ± 6.5^a^	22.5 ± 0.1^b^	24.1 ± 0.8^c^
	FEDEARROZ68	NBF2P	15.0 ± 1.9^ef^	10.0 ± 0.3 ^cd^	10.6 ± 0.3^b^	68.8 ± 5.5^a^	89.4 ± 3.9^ab^	19.5 ± 0.2^d^	24.0 ± 2.0 ^cd^
	Average Palmira		17.2 ± 3.1^B^	10.8 ± 1.5^A^	10.4 ± 0.7^A^	61.1 ± 10.2^A^	82.2 ± 8.4^A^	22.1 ± 2.0 ^A^	24.8 ± 2.4^A^
Santa Rosa	BRRI Dhan64	BF1SR	31.6 ± 0.6^b^	11.2 ± 1.2^bc^	6.9 ± 0.0^f^	21.8 ± 0.4^e^	52.9 ± 5.6^e^	21.8 ± 1.3^bc^	26.5 ± 0.2^b^
	BF14AR035	BF2SR	34.6 ± 0.8^ab^	11.2 ± 0.2^bc^	9.2 ± 0.1^d^	26.4 ± 0.7^de^	69.7 ± 0.7 ^cd^	23.8 ± 0.1^a^	29.1 ± 0.3^a^
	BF14AR050	BF3SR	35.1 ± 0.8^a^	12.3 ± 0.2^ab^	8.2 ± 0.1^e^	23.3 ± 0.6^de^	54.0 ± 5.4^de^	24.1 ± 0.2^a^	22.8 ± 1.8^d^
	IR64	NBF1SR	25.7 ± 0.8^c^	8.7 ± 0.4^ef^	8.0 ± 0.1^e^	31.1 ± 1.7^de^	73.2 ± 11.3^bc^	23.5 ± 0.6^ab^	24.3 ± 0.5^c^
	FEDEARROZ68	NBF2SR	25.3 ± 1.2^c^	7.9 ± 0.5^f^	8.2 ± 0.1^e^	32.3 ± 0.6^d^	88.0 ± 6.1^ab^	21.1 ± 0.2^c^	28.7 ± 0.9^a^
	Average Santa Rosa		30.5 ± 4.7^A^	10.3 ± 1.9^A^	8.1 ± 0.8^B^	27.0 ± 4.6^B^	67.6 ± 14.6^B^	22.9 ± 1.3^A^	26.3 ± 2.7^A^
	Average BF		26.5 ± 8.2^A^	11.6 ± 1.0^A^	9.3 ± 1.6^A^	39.1 ± 17.3^B^	67.7 ± 11.8^B^	23.1 ± 1.5^A^	25.7 ± 2.9^A^
	Average NBF		19.9 ± 6.6^B^	9.0 ± 0.9^B^	9.2 ± 1.3^A^	51.4 ± 22.8^A^	85.6 ± 8.4^A^	21.7 ± 1.7^A^	25.3 ± 2.3^A^

¥ BF = biofortified entry. NBF = non-biofortified entry. BF1P, BF2P, BF3P, BF1SR, BF2SR and BF3SR = biofortified entries. NBF1P, NBF2P, NBF1SR and NBF2 SR = non-biofortified entries. Values for each grain entry are given as mean ± standard deviation of 3 processing batch repetitions. Different lowercase letters within each column indicate significant differences between entries (*p* < 0.05). Different uppercase letters within each column indicate significant differences between locations and between rice type (*p* < 0.05).

### 2.2 Paddy soaking and steaming

A traditional method of single boiled process where paddy is soaked in water at room temperature and steamed for a short period of time was used with modifications based on paddy properties (Larsen et al., 2000). Three batches of paddy rice for each of the five entries harvested in each location were soaked to obtain a total of 30 soaking batches. For each batch, 2000 g of RAW were soaked in 2600 mL of distilled milli-Q water at 20 °C for 40 h to reach moisture content of 33 ± 2% (Supplementary Fig. 1). For steaming, half of the soaked paddy from each batch was put in a food steamer (Model 5712, Oster Electrics, USA) and steamed for 13 min (PB13), until the husk of most of the paddy grains was slightly open. After steaming, the paddy was kept in a bulk until the temperature of steamed rice dropped slowly to <50 °C simulating what typically occurs with small commercial parboiling facilities in South Asia. The other half of soaked paddy from each batch was steamed for 16 min (PB16) and cooled down immediately after steaming in a clean plastic surface. The 13**–**16 min steaming range was used to ensure that the grain was sufficiently steamed to prevent excessive occurrence of underparboiled kernels and to prevent excessive occurrence of overparboiled kernels. Steamed paddy was dried in a forced air oven (Model FD23, Binder, Germany) with temperature of 30 °C. Paddy was first dried to 18.0**–**22.0% moisture and tempered for 4**–**6 h. After tempering, paddy was further dried to 11.0**–**13.0% moisture content.

### 2.3 Milling

The 30 batches of RAW, 30 batches of PB13 and 30 batches of PB16 were dehulled in a rubber dehuller to obtain brown rice identified as NPBDOM0, PB13DOM0 and PB16DOM0, respectively. Brown rice was cleaned to remove husk residues and broken rice. Subsamples of each batch of NPBDOM0 and PB13DOM0 were then milled at 7.5% and 10.0% ± 0.5% degrees of milling (DOM) to obtain NPBDOM7.5, NPBDOM10, PB13DOM7.5 and PB13DOM10, whereas NPB16DOM0 was milled only at 7.5% ± 0.5% DOM to obtain PB16DOM7.5 (Supplementary Fig. 1). The DOM or percentage of bran removed (pericarp + most of the germ + most of the aleurone) was determined by measuring the weight of the grain before and after milling and such difference was expressed as a percentage of the grain weight before milling. For each DOM, 3**–**6 milling batches of 29.00 ± 0.01 g of brown rice were milled. The three milled batches that were the closest to the target DOM and no more than 0.5% off were selected for analysis. After milling, broken grain was separated and only head rice was used for analysis. Zn, Fe and PA was analyzed for all brown and milled samples of parboiled and non-parboiled rice.

### 2.4 Zn and Fe analysis

An Oxford X-Supreme 8000 energy-dispersive X-ray fluorescence spectrometer (XRF) (Oxford Instruments, UK) was used to measure Zn concentration in non-parboiled (NPBDOM0, NPBDOM7.5, NPBDOM10) and parboiled (PB13DOM0, PB13DOM7.5, PB13DOM10, PB16DOM0 and PB16DOM7.5) rice, as described by Paltridge et al. (2012). After the analysis by XRF, an inductively coupled plasma optical emission spectrometer (ICP-OES) was used to measure Zn and Fe concentration in the same samples following the method described by Wheal et al. (2011). The Zn and Fe concentrations presented in all tables and figures were obtained by ICP-OES. The limit of detection and quantification for Zn was <0.01 µg.g^−1^ and 0.02 µg.g^−1^ whereas for Fe it was <0.01 µg.g^−1^ and <0.01 µg.g^−1^, respectively. All analysis results of the certified standard were within the certified acceptable range of 23.1 ± 0.9 µg.g^−1^ for Zn and 11.4 µg.g^−1^ ± 0.8 µg.g^−1^ for Fe and with a relative standard deviation of 1.5% for Zn and 3.9% for Fe.

### 2.5 True Zn, Fe and PA retention

Percentage of true retention (TR) was calculated as follows:

$$NutrientTR\%=\frac{Nutrientcontentpergofprocessedrice\left(drybasis\right)*gofriceafterprocessing}{Nutrientcontentpergofbrownricebeforeprocessing\left(drybasis\right)*gofbrownricebeforeprocessing}$$

TR percentage values were used to determine the total proportion of Zn, Fe and PA lost during processing. TR is useful to understand how much the edible part of the rice crop could impact the intake of each nutrient based on the expected amount of crop available per person.

### 2.6 PA analysis

The total PA content was analyzed by the Latta and Eskin (1980) method with modifications. For each sample, 1 g of ground sample was weighed and 20 mL of 0.65 M HCl were added. Extraction was performed for 2 h in multivortex. It was then centrifuged for 15 min at 3800 rpm. 5 mL of the supernatant was taken and introduced into polyprep chromatographic columns (Bio-Rad Laboratories, USA) containing an AG-1-X8 anion exchange resin (100**–**200 mesh, chloride form, 0.8 × 4 cm) to separate PA from the sample extract. The interfering compounds and inorganic phosphorus were removed by washing with 5 mL of 18 MΩ.cm water followed by 10 mL of 0.07 M NaCl. The PA bound to the resin was eluted with 30 mL of 0.7 M NaCl. An aliquot of the eluate (0.9 mL) was vortexed with 0.3 mL of Wade reagent (0.03% Iron Chloride (III), 0.3% sulfosalicylic acid). The absorbance of the salicylate-Fe (III) complex was determined on a spectrophotometer (BioTek Instruments, Inc., USA) at 500 nm. The PA concentration was calculated from a standard curve (0–60 g L^-1^) that was obtained with PA dipotassium salt (Sigma-Aldrich, Canada). The limit of detection and quantification for PA was 0.6 mg.g^−1^ and 1.3 mg.g^−1^. The percentage of recovery was 93.7, 104.7 and 96.8, for low, medium and high levels of the working curve, respectively (SD ± 1.95%).

### 2.7 Data analysis

To evaluate the effect of location and entry in Zn, Fe and PA concentration, a 2-factor analysis of variance model was used for each parboiling and milling type (NPBDOM0, NPBDOM7.5, NPBDOM10, PB13DOM0, PB13DOM7.5, PB13DOM10, PB16DOM0 and PB16DOM7.5). The effect processing step (parboiling and non-parboiling) and grain type (biofortified and non-biofortified) on Zn, Fe and PA TR was evaluated using a 2-factor analysis of variance model. The effect of processing on Zn, Fe and PA TR for each entry was evaluated using a one-way analysis of variance. Analysis of variance was done using the GLM procedure in SAS 9.4 (SAS Institute, Cary, NC). Means separation was calculated using PLM procedure and Tukey-Kramer multiple comparison method. Differences between means were considered significant at *p* < 0.05. To evaluate the parboiling and milling effect, Zn, Fe and PA concentration and TR was expressed as mean ± standard deviation of three parboiling repetitions for each entry.

## 3 Results and discussion

### 3.1 Grain characterization

Brown rice weight ranged from 19.5 to 24.7 g per 1000 grains. Length (5.6–7.4 mm) and width (2.2–2.7 mm) of NPBDOM0 resulted in length to width ratios of 2.1–3.2. Amylose content of grain of BF entries (BF1, BF2 and BF3) was 22.0–29.1% and NBF varieties (NBF1 and NBF2) was 24.0–28.7% (Table 1). Both groups had grain classified with medium and slender shape and as intermediate and high amylose rice, which are types generally accepted in countries were parboiled rice is commonly consumed (Calingacion et al., 2014). Ether extract of NBPDOM7.5 was 0.9 ± 0.2% and whiteness of PB13DOM7.5 was 21.9–31.0% (Supplementary Table 1).

### 3.2 Zn, Fe and PA concentration in non-parboiled brown rice (NPBDOM0)

The range of Zn and Fe concentration in NPBDOM0 was 13.4–35.1 µg.g^−1^ and 7.9–13.2 µg.g^−1^, respectively (Table 1). These concentrations were within the values (13.5–58.4 µg.g^−1^ for Zn and 7.5–24.4 µg.g^−1^ for Fe) reported for 939 genotypes in brown rice evaluated at the International Rice Research Institute in the Philippines (Welch & Graham, 2002). BF entries had higher Zn concentration than NBF entries with an average of 26.5 µg.g^−1^ and 19.9 µg.g^−1^, respectively, except for BF1P, which was similar to NBF2P (Table 1). BF rice had 35.2% higher Zn concentration than NBF rice in Palmira and 32.4% in Santa Rosa. The higher Zn concentration in BF entries within the same location was expected because these entries were selected for their higher ability to accumulate Zn regardless of the environmental conditions. Across locations, Fe concentration was also higher in BF entries (11.6 µg.g^−1^) compared to NBF entries (9.0 µg.g^−1^), however, the magnitude of the difference was lower than for Zn and no difference was found within Palmira location for NBF2P and the BF lines (Table 1).

Average Zn concentration in NPBDOM0 from rice grown in Santa Rosa (30.5 µg.g^−1^) was 77% higher compared to Palmira (17.2 µg.g^−1^) (Table 1). In contrast, average Fe concentration did not differ between locations (10.8 and 10.3 µg.g^−1^ for Palmira and Santa Rosa, respectively), except for NBF2P, which was higher than NBF2SR (Table 1). The differences in agronomic management could partially explain the lower Zn concentration in all rice grown at the CIAT station in Palmira where soil was saturated with water during all stages of plant growth and pH was high (7.7), whereas in Santa Rosa, rice plants were grown under aerobic conditions with soil pH of 5.5. Although differences in soil water management and soil pH could partially explain the difference in Zn accumulation in grain among sites, other factors such as chemical availability in the rhizosphere induced by plant roots and the increased acquisitions area by root growth or mycorrhizae are known to impact Zn acquisition by plants (Gao et al., 2012). Saenchai et al. (2016) reported up to 6 µg.g^−1^ higher zinc concentration in rice cultivated in soils with high Zn availability compared to rice cultivated in soils with low Zn availability. Mayer et al. (2008) observed that Zn concentration in brown rice produced under soils with low Zn availability in Bangladesh was 18.6 µg.g^−1^ in the irrigated season and 20.8 µg.g^−1^ in monsoon season, similar to the Zn levels in NPBDOM0 produced in Palmira.

The Zn concentration in brown rice (BF and NBF) did correlate with its Fe concentration within each location (R^2^ = 0.65 in Palmira and 0.86 in Santa Rosa) (Supplementary Fig. 2A and B). The correlation was previously observed and attributed to co-localization of QTL’s for Zn and Fe accumulation in rice grain (Swamy et al., 2018).

PA concentration in NPBDOM0 ranged 6.9–11.6 mg.g^−1^. Contrary to Zn and Fe, PA in NPBDOM0 of BF rice (9.3 mg.g^−1^) was similar to NBF rice (9.2 mg.g^−1^) (Table 1). Similar PA concentration between BF and NBF was expected since the correlation between Zn and PA accumulation in rice appears to be poor (Stangoulis et al., 2007). However, NPBDOM0 obtained from rice harvested in Palmira had higher PA (10.4 mg.g^−1^) than rice harvested in Santa Rosa (8.1 mg.g^−1^), except for BF2SR (Table 1). The higher PA in brown rice from Palmira could be due to the water saturated soil during all stages of plant growth and high pH at this location, such soil conditions are adequate for phosphorus absorption by rice plants (Perera et al., 2018). PA concentration in brown rice was similar to the 6.8–10.3 mg.g^−1^ previously reported for 72 cultivars (Liu et al., 2005) and 7.2–11.9 mg.g^−1^ found in 56 genotypes from China (Liang et al., 2007).

The average PA:Zn ratio in NPBDOM0 was lower among BF (39.1) than NBF (51.4) rice (Table 1). BF rice grown in Palmira had lower PA:Zn ratio than NBF rice, but for rice from Santa Rosa, significant difference was only found between biofortified BF1SR and non-biofortified NBF2SR rice (Table 1). Average PA:Zn ratio was higher in brown rice from Palmira (61.1) compared to Santa Rosa (27.0), due to both the higher accumulation of PA and lower accumulation of Zn in rice grown in Palmira (Table 1). In general, average PA:Fe ratio was lower among BF (67.7) than NBF (85.6) entries (Table1). Lower PA:Zn and PA:Fe ratios found in biofortified entries could be a desired attribute for biofortified brown rice since higher ratios are associated with lower absorption of such minerals (Hambidge et al., 2008, Al Hasan et al., 2016).

### 3.3 Zn, Fe and PA retention in brown parboiled rice (PB13DOM0 and PB16DOM0)

**Fig. 1:**
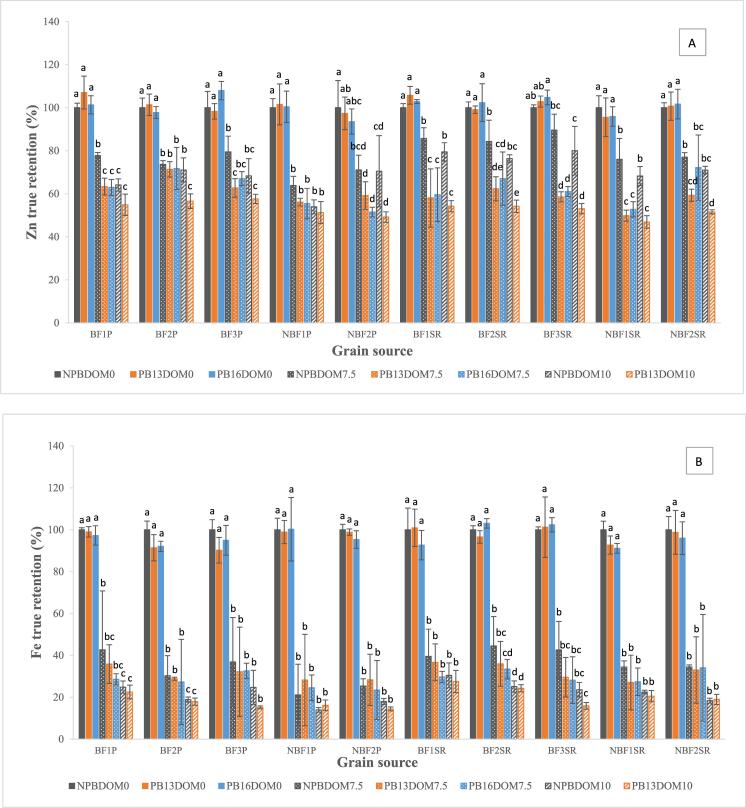

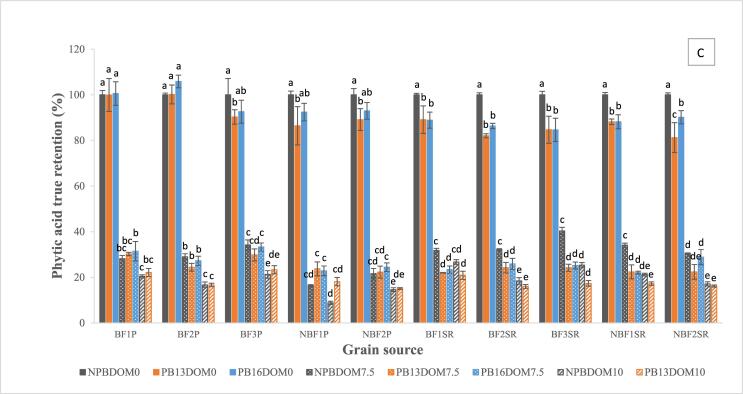
Zn (A), Fe (B) and PA (C) true retention in non-parboiled (NPB) and parboiled (PB13 and PB16) brown (DOM0) and milled rice (DOM7.5 and DOM10) of three biofortified (BF1, BF2 and BF3) and two non-biofortified (NBF1 and NBF2) entries evaluated at two locations (Palmira = P and Santa Rosa = SR) in Colombia. Bars with different letters within each grain source are statistically different (*p* < 0.05).

**Fig. 2:**
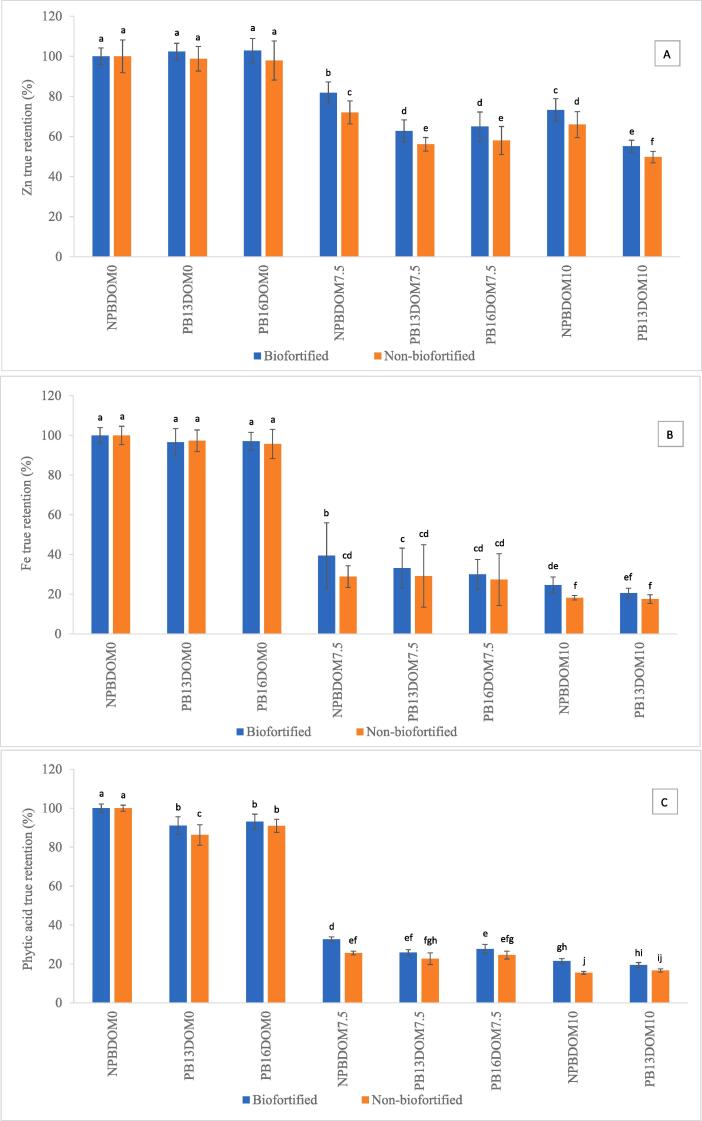
Zn (A), Fe (B) and PA (C) true retention in non-parboiled brown rice (NPBDOM0), milled non-parboiled rice (NPBDOM7.5 and NPBDOM10), brown parboiled rice (PB13DOM0 and PB16DOM0) and milled parboiled rice (PB13DOM7.5, PB16DOM7.5 and PB13DOM10). Average of five entries and two locations in Colombia. Bars with different letters are statistically different (*p* < 0.05).

Zn and Fe concentration in brown parboiled rice ranged 13.5–36.8 µg.g^−1^ and 7.6–12.6 µg.g^−1^ (Table 2, Table 3), representing 93.6–108.0% and 91.1–103.0% TR, respectively (Fig. 1A and B). Similar to NPBDOM0, brown parboiled rice of BF entries had higher Zn and Fe concentration compared to NBF (Table 2, Table 3). In general, both parboiling methods did not affect Zn and Fe TR in brown rice (Fig. 2A and B), suggesting that Zn and Fe from the husk did not mobilize in significant amounts to the grain (brown rice) during parboiling either with low steaming time (PB13) or high steaming time (PB16).

**Table 2:** Zn concentration in brown parboiled, milled non-parboiled and milled parboiled rice grain of three biofortified and two non-biofortified entries evaluated at two locations in Colombia¥.

Location	Grain source code	PB13DOM0	PB16DOM0	NPBDOM7.5	PB13DOM7.5	PB16DOM7.5	NPBDOM10	PB13DOM10
		Zn (µg.g^−1^)	Zn (µg.g^−1^)	Zn (µg.g^−1^)	Zn (µg.g^−1^)	Zn (µg.g^−1^)	Zn (µg.g^−1^)	Zn (µg.g^−1^)
Palmira	BF1P	18.2 ± 0.9^d^	17.2 ± 0.4 ^fg^	14.2 ± 0.2^de^	11.6 ± 0.4^de^	11.5 ± 0.4^de^	12.1 ± 0.8 ^cd^	10.4 ± 1.0^c^
	BF2P	20.9 ± 1.3 ^cd^	20.2 ± 0.8^ef^	15.9 ± 0.3^de^	15.4 ± 0.6^c^	15.5 ± 1.5 ^cd^	15.9 ± 0.5^bc^	12.6 ± 0.8^b^
	BF3P	20.1 ± 1.9^d^	22.1 ± 0.8^de^	17.5 ± 1.3 ^cd^	13.7 ± 0.6 ^cd^	14.7 ± 0.5^d^	15.5 ± 0.8^bc^	13.1 ± 0.5^b^
	NBF1P	13.6 ± 0.9^e^	13.5 ± 1.4 ^h^	9.2 ± 0.4^f^	8.1 ± 0.1^f^	8.0 ± 0.6^e^	8.0 ± 0.2^d^	7.6 ± 0.5^d^
	NBF2P	14.5 ± 1.0^e^	14.0 ± 1.4^gh^	11.4 ± 0.8^ef^	9.5 ± 0.6^ef^	8.2 ± 0.2^e^	11.5 ± 1.5 ^cd^	8.2 ± 0.7^d^
	Average Palmira	17.5 ± 3.3^B^	17.4 ± 3.7^B^	13.6 ± 3.4^B^	11.7 ± 3.0^B^	11.6 ± 3.5^B^	12.6 ± 3.2^B^	10.4 ± 2.5^B^
Santa Rosa	BF1SR	33.4 ± 1.3^a^	32.5 ± 0.7^b^	29.1 ± 1.4^b^	19.7 ± 2.7^b^	20.3 ± 2.5^abc^	27.9 ± 1.0^a^	19.0 ± 0.8^a^
	BF2SR	34.4 ± 1.8^a^	35.5 ± 1.8^ab^	31.3 ± 3.1^ab^	23.2 ± 1.3^a^	24.9 ± 3.1^a^	29.4 ± 1.3^a^	20.8 ± 0.6^a^
	BF3SR	36.1 ± 0.6^a^	36.8 ± 1.7^a^	33.8 ± 2.5^a^	22.1 ± 0.5^ab^	23.0 ± 0.5^ab^	31.2 ± 4.2^a^	20.7 ± 0.9^a^
	NBF1SR	24.5 ± 0.9^bc^	24.6 ± 0.5 ^cd^	21.0 ± 2.0^c^	13.7 ± 0.4 ^cd^	14.5 ± 0.5^d^	19.4 ± 0.3^b^	13.4 ± 0.8^b^
	NBF2SR	25.4 ± 1.3^b^	25.7 ± 1.2^c^	20.9 ± 0.4^c^	16.1 ± 0.5^c^	19.7 ± 3.0^bc^	19.9 ± 0.8^b^	14.5 ± 0.6^b^
	Average Santa Rosa	30.8 ± 5.4^A^	31.0 ± 5.6^A^	27.2 ± 6.0^A^	19.0 ± 4.0^A^	20.5 ± 3.9^A^	25.5 ± 5.5^A^	17.7 ± 3.5^A^
	Average BF	27.2 ± 8.2^A^	27.4 ± 8.5^A^	23.6 ± 8.7^A^	17.6 ± 4.7^A^	18.3 ± 5.2^A^	22.0 ± 8.4^A^	16.1 ± 4.6^A^
	Average NBF	19.5 ± 6.3^B^	19.4 ± 6.6^B^	15.6 ± 6.2^B^	11.9 ± 3.7^B^	12.6 ± 5.6^B^	14.7 ± 5.9^B^	10.9 ± 3.5^B^

¥ BF = biofortified entry. NBF = non-biofortified entry. Values for each grain source are given as mean ± standard deviation of 3 processing batch repetitions. Different lowercase letters within the same column indicate significant differences between entries (*p* < 0.05). Different uppercase letters within each column indicate significant differences between locations and between rice type (*p* < 0.05). PB13DOM0 = brown parboiled steamed for 13 min, PB16DOM0 = brown parboiled steamed for 16 min, NPBDOM7.5 = milled non-parboiled (7.5% degrees of milling), PB13DOM7.5 = parboiled steamed for 13 min and milled at 7.5% degrees of milling, PB16DOM7.5 = parboiled steamed for 16 min and milled at 10% degrees of milling, NPBDOM10 = milled non-parboiled (10% degrees of milling) and PB13DOM10 = parboiled milled at 10% degrees of milling.

**Table 3:** Fe concentration in brown parboiled, milled non-parboiled and milled parboiled rice grain of three biofortified and two non-biofortified entries evaluated at two locations in Colombia¥.

Location	Grain source code	PB13DOM0 Fe (µg.g^−1^)	PB16DOM0 Fe (µg.g^−1^)	NPBDOM7.5 Fe (µg.g^−1^)	PB13DOM7.5 Fe (µg.g^−1^)	PB16DOM7.5 Fe (µg.g^−1^)	NPBDOM10 Fe (µg.g^−1^)	PB13DOM10 Fe (µg.g^−1^)
Palmira	BF1P	10.4 ± 0.4^abc^	10.2 ± 0.4^c^	4.8 ± 1.4^abc^	4.0 ± 0.4^abc^	3.2 ± 0.1^ab^	2.9 ± 0.3^abc^	2.6 ± 0.4^bc^
	BF2P	10.3 ± 0.2^abc^	10.4 ± 0.2^bc^	3.6 ± 0.3^bcd^	3.5 ± 0.0^abcd^	3.3 ± 0.7^ab^	2.3 ± 0.1^abc^	2.2 ± 0.1 ^cd^
	BF3P	11.9 ± 0.3^a^	12.5 ± 0.4^a^	5.2 ± 1.1^ab^	4.5 ± 1.0^a^	4.6 ± 0.2^a^	3.7 ± 1.3^ab^	2.2 ± 0.1 ^cd^
	NBF1P	9.1 ± 0.0^bc^	9.2 ± 0.9 ^cd^	2.1 ± 0.3^d^	2.8 ± 0.6 ^cd^	2.4 ± 0.1^b^	1.4 ± 0.1^c^	1.7 ± 0.4^d^
	NBF2P	9.9 ± 0.4^abc^	9.6 ± 0.5^c^	2.7 ± 0.1^d^	3.0 ± 0.4^bcd^	2.5 ± 0.4^ab^	2.0 ± 0.1^bc^	1.6 ± 0.1^d^
	Average Palmira	10.3 ± 1.0^A^	10.4 ± 1.3^A^	3.7 ± 1.3^A^	3.6 ± 0.7^A^	3.2 ± 0.9^A^	2.5 ± 0.9^A^	2.1 ± 0.4^A^
Santa Rosa	BF1SR	11.3 ± 2.0^ab^	10.3 ± 0.3^bc^	4.8 ± 0.6^abc^	4.4 ± 0.4^ab^	3.5 ± 0.1^ab^	3.7 ± 0.4^a^	3.4 ± 0.4^a^
	BF2SR	10.7 ± 0.3^abc^	11.7 ± 0.3^ab^	5.3 ± 0.7^ab^	4.3 ± 0.5^ab^	4.0 ± 0.2^ab^	3.1 ± 0.4^ab^	3.0 ± 0.3^ab^
	BF3SR	12.5 ± 1.6^a^	12.6 ± 0.3^a^	5.7 ± 0.8^a^	3.9 ± 0.4^abcd^	3.7 ± 0.4^ab^	3.2 ± 0.5^ab^	2.2 ± 0.2 ^cd^
	NBF1SR	8.0 ± 0.1^c^	7.9 ± 0.2^de^	3.2 ± 0.1 ^cd^	2.5 ± 0.3^d^	2.5 ± 0.2^ab^	2.2 ± 0.1^bc^	2.0 ± 0.3 ^cd^
	NBF2SR	7.8 ± 0.4^c^	7.6 ± 0.4^e^	2.9 ± 0.0 ^cd^	2.8 ± 0.4 ^cd^	2.9 ± 0.7^ab^	1.6 ± 0.1^c^	1.7 ± 0.1^d^
	Average Santa Rosa	10.1 ± 2.1^A^	10.0 ± 2.2^A^	4.4 ± 1.2^A^	3.6 ± 0.9^A^	3.3 ± 0.6^A^	2.8 ± 0.9^A^	2.4 ± 0.7^A^
	Average BF	11.2 ± 0.9^A^	11.3 ± 1.1^A^	4.9 ± 0.7^A^	4.1 ± 0.4^A^	3.7 ± 0.5^A^	3.2 ± 0.5^A^	2.6 ± 0.5^A^
	Average NBF	8.7 ± 1.0^B^	8.6 ± 1.0^B^	2.7 ± 0.5^B^	2.8 ± 0.2^B^	2.6 ± 0.2^A^	1.8 ± 0.3^B^	1.7 ± 0.2^B^

¥ BF = biofortified entry. NBF = non-biofortified entry. Values for each grain source are given as mean ± standard deviation of 3 processing batch repetitions. Different lowercase letters within each column indicate significant differences between entries (*p* < 0.05). Different uppercase letters within each column indicate significant differences between locations and between rice type (*p* < 0.05). PB13DOM0 = brown parboiled steamed for 13 min, PB16DOM0 = brown parboiled steamed for 16 min, NPBDOM7.5 = milled non-parboiled (7.5% degrees of milling), PB13DOM7.5 = parboiled steamed for 13 min and milled at 7.5% degrees of milling, PB16DOM7.5 = parboiled steamed for 16 min and milled at 10% degrees of milling, NPBDOM10 = milled non-parboiled (10% degrees of milling) and PB13DOM10 = parboiled milled at 10% degrees of milling.

Parboiling decreased PA TR in brown rice of most entries (81.2–105.9%) (Fig. 1C), resulting in PA concentrations ranging 6.2–10.7 mg.g^−1^ in PB13DOM0 and PB16DOM0 (Supplementary Table 2). However, no difference was observed between PA concentration of BF and NBF rice (Supplementary Table 2). The reduction in PA concentration in parboiled rice could have occurred due the degradation of PA by endogenous phytases activated during soaking process and leaching into the soaking water, as reported in earlier studies with brown rice (Liang et al., 2009). Similar to non-parboiled rice, average PA in brown parboiled rice from Palmira (9.9 mg.g^−1^) was higher than from Santa Rosa (7.0 mg.g^−1^) (Supplementary Table 2). In general, no difference was found between the average PA:Zn ratios in BF and NBF within each location, except for BF2P and BF3P which were lower than NBF entries when subject to NPB16DOM0. However, the average PA:Zn ratio was higher in brown parboiled rice from Palmira (58.1) compared to Santa Rosa (23.2) due to the higher Zn concentration in rice from Santa Rosa (Supplementary Table 3). Average PA:Fe ratio in brown parboiled rice from Palmira was also higher (81.8) than rice from Santa Rosa (61.1) due to the lower PA content in samples from Santa Rosa (Supplementary Table 4).

### 3.4 Zn, Fe and PA retention in milled non-parboiled rice (NPBDOM7.5)

The range of Zn and Fe in NPBDOM7.5 was 9.2–33.8 µg.g^−1^ and 2.1–5.7 µg.g^−1^, respectively (Table 2, Table 3). The average Zn concentration in BF entries (23.6 µg.g^−1^) was higher than NBF entries (15.6 µg.g^−1^) (Table 2), which also resulted in higher TR for BF (81.8%) compared to NBF (72.0%) (Fig. 2A). For all entries, average Zn concentration in NPBDOM7.5 rice from Santa Rosa (27.2 µg.g^−1^) was higher than Palmira (13.6 µg.g^−1^) (Table 2). Furthermore, Zn TR values of rice entries grown in Santa Rosa (76.0–89.6%) were higher than rice grown in Palmira (63.8–79.5%) (Fig. 1A). Zn TR values for NPBDOM7.5 rice grown in Palmira were similar than the 61–76% found by Mayer et al. (2008) for 33 rice samples milled at commercial facilities without DOM reported.

In general, average Fe concentration in NPBDOM7.5 of BF entries (4.9 µg.g^−1^) was higher than NBF entries (2.7 µg.g^−1^) (Table 3), representing 39.4% and 28.9% TR, respectively (Fig. 2B). Fe concentration in NPBDOM7.5 was within the lower range of values reported by Jiang et al. (2007) after milling 274 genotypes (1.0–26.8 µg.g^−1^). Fe concentration in NPBDOM7.5 from Santa Rosa and from Palmira was similar (Table 3), resulting in 39.4% and 28.9% TR, respectively (Fig. 2B).

Higher mass losses for Zn (10.4–36.2%) and Fe (55.5–78.9%) compared to the 7.5% of grain mass removed during milling (DOM7.5) indicated that the concentration of these minerals were higher in the outer layers removed during the milling process than in the endosperm. In concordance with these results, previous studies have shown that rice bran contains up to 25% of the total Zn mass in rice grain due to the higher concentration of Zn in the germ and pericarp (Liang et al., 2008, Lu et al., 2013, Oli et al., 2016, Jo and Todorov, 2019). Zn retention after milling was higher than Fe retention for all genotypes, confirming that concentration of Zn in the grain of biofortified rice was more uniformly distributed compared to Fe, as previously reported for non-biofortified rice (Hansen et al., 2012, Jo and Todorov, 2019). Despite the Zn losses during milling, BF entries had lesser Zn losses than NBF entries, suggesting that Zn in the BF entries was more concentrated in the endosperm, which could result in higher Zn intake. Even though Fe concentration in the BF milled non-parboiled rice was lower than Zn, the additional Fe provided by this type of rice product could contribute to increase the Fe intake of deficient populations that consume non-parboiled rice.

PA concentration in NPBDOM7.5 was 1.8–4.3 mg.g^−1^ (Supplementary Table 2). Average PA TR in BF (32.6%) and NBF (25.6%) was lower than in brown rice (Fig. 2C). In general, NPBDOM7.5 from grains collected in Palmira and Santa Rosa had similar concentrations (2.9 and 3.0 mg.g^−1^, respectively) (Supplementary Table 2). The low PA TR in milled non-parboiled rice was expected because it is known that PA is mostly located in the outer layers of the rice grain (Liang et al., 2008) which were removed during milling. Average PA:Zn ratio in NPBDOM7.5 was similar between BF (15.4) and NBF (16.8) rice across locations but it was higher in rice from Palmira (20.9) compared to Santa Rosa (10.9) due to the lower Zn concentration in NPBDOM7.5 from Palmira (Supplementary Table 3). The PA:Fe ratio in BF rice was higher than in NBF rice for samples from Santa Rosa but no such difference was found in Palmira (Supplementary Table 4).

### 3.5 Zn, Fe and PA retention in milled parboiled rice (PB13DOM7.5, PB16DOM7.5 and PB13DOM10)

The range of Zn and Fe concentration in milled parboiled rice at 7.5 DOM was 8.0–24.9 µg.g^−1^ and 2.4–4.6 µg.g^−1^, respectively (Table 2, Table 3). The average Zn concentration in milled parboiled rice of BF entries (18.0 µg.g^−1^) was higher than NBF entries (12.3 µg.g^−1^) (Table 2), resulting in 58.1–71.8% and 49.8–72.2% TR, respectively (Fig. 1A). Zn TR in milled parboiled rice was similar between rice grown in Palmira (51.5–71.8% TR) and Santa Rosa (49.8–72.2% TR) despite the large difference in Zn concentration between these two locations (11.7 µg.g^−1^ and 19.8 µg.g^−1^, respectively) (Fig. 1A and Table 2). In general, Zn TR in milled parboiled rice was lower than in milled non-parboiled rice (Fig. 2A).The average Fe concentration in milled parboiled rice at 7.5% DOM of most BF entries was higher than in NBF varieties with 3.9 µg.g^−1^ (31.6% TR) and 2.7 µg.g^−1^ (28.3% TR), respectively (Table 3 and Fig. 2B). However, no difference was found in Fe TR between parboiled and non-parboiled rice (Fig. 2B). Fe TR in milled parboiled rice from Santa Rosa (27.0–36.7%) was not different from Palmira (23.4–35.8%), and in general were lower that Fe TR in non-parboiled and parboiled brown rice (Fig. 1B).

PA in PB13DOM7.5 and PB16DOM7.5 rice ranged 1.6–4.1 mg.g^−1^ (Supplementary Table 2). In general, no difference was found in PA TR between BF (26.8%) and NBF rice (23.6%) (Fig. 2C), however the TR for each entry was significantly lower than brown rice (Fig. 1C). PA:Zn ratio was similar between NBF (21.7) and BF (17.0) in PB13DOM7.5 and PB16DOM7.5 but was higher in most rice from Palmira (26.9) compared to Santa Rosa (11.0) (Supplementary Table 3), as a result of the lower Zn concentration in milled parboiled rice from Palmira.

Concentration of Zn, Fe and PA was also determined in non-parboiled and parboiled rice milled at a higher DOM (NPBDOM10 and PB13DOM10) (Table 2, Table 3 and Supplementary Table 2). In NPBDOM10, Zn concentration ranged 8.0–31.2 µg.g^−1^ (Table 2), resulting in Zn TR of 54.0–80.0% which was lower than non-parboiled and parboiled brown rice (Fig. 1A). In PB13DOM10 Zn concentration ranged 7.6–20.8 µg.g^−1^, representing Zn TR of 46.9–57.6% which in general was lower than all brown rice, all milled rice 7.5% DOM and non-parboiled rice at 10% DOM within each BF and NBF groups (Table 2 and Fig. 1A). Fe concentration in NPBDOM10 and PB13DOM10 ranged 1.4–3.7 µg.g^−1^ and 1.6–3.4 µg.g^−1^, respectively (Table 3), representing 14.0–30.5% TR for non-parboiled and 14.4–27.5% TR for parboiled rice (Fig. 1B). Fe TR in NPBDOM10 was lower in NBF than in BF rice but was similar between NBF and BF in PBDOM10 (Fig. 2B). Fe concentration in PB13DOM10 was lower than brown rice and rice milled at 7.5% DOM (Table 3). PA concentration in rice milled at 10% DOM was 1.0–2.7 mg.g^−1^ (8.8–26.7% TR) in non-parboiled rice and 1.5–3.0 mg.g^−1^ (15.0–23.3%) in parboiled rice (Supplementary Table 2 and Fig. 1C).

For parboiled and non-parboiled rice, Zn, Fe and PA true retention in rice milled at 10% DOM was generally lower than rice milled at 7.5% DOM within each BF and NBF group (Fig. 2A–C). Contrary to Zn TR, in most cases Fe and PA TR in milled parboiled rice was similar to milled non-parboiled rice for each DOM. The similar Fe and PA TR between parboiled and non-parboiled rice meant that there was no additional accumulation of Fe or PA during soaking or steaming of rice. Consequently, parboiling did not increase Fe content or reduce the antinutrient PA in milled rice.

On the other hand, milled parboiled rice had lower Zn concentration than milled non-parboiled rice. Both of these processed forms of rice had lower Zn concentration compared to brown rice because of Zn lost during the removal of the pericarp and germ. However, parboiling exacerbated the zinc losses during milling. The lower Zn TR of parboiled rice milled at 7.5% and 10% DOM compared to milled non-parboiled rice found in this study is similar to the findings of Denardin et al. (2004). The low Zn TR in milled parboiled rice contrasts with the high thiamine and riboflavin TR found in milled parboiled rice compared to non-parboiled rice (Grewal & Sangha, 1990). The lower Zn TR in parboiled milled rice could be due to the binding of Zn with PA present in the outer layers during mobilization of Zn within the grain during parboiling. PA binds with Zn and other minerals such as Ca and Mg in an aqueous matrix (Chan, 1988, Maenz et al., 1999). The high moisture content of the grain during soaking and high temperature during steaming could facilitate the binging of Zn to PA in rice. Oli et al. (2016) observed that the Zn layer within the bran layer of rice grain was broader after parboiling which they attributed to the inward diffusion of Zn from the bran layer. However, part of the reason why the Zn layer of the bran increase after parboiling (soaking and steaming) could be due to the movement and potential binding of Zn from the endosperm to the outer layers as suggested by the lower Zn concentration found in milled parboiled samples compared to the milled non-parboiled samples evaluated in this study.

### 3.6 Rapid measurement of Zn in rice

An XRF rapid method to measure Zn was used to analyze all samples. Zn concentration measured by XRF highly correlated (R^2^ = 0.97) to the values obtained by the reference method (ICP-OES) (Supplementary Fig. 3). Paltridge et al. (2012) reported that XRF could be used to measure Zn in raw rice, but up to date the use of XRF has not been evaluated for parboiled rice. The results in this study suggests that Zn concentration could be estimated by XRF not only in raw but also in parboiled rice.

### 3.7 Contribution to Zn estimated average requirement (EAR)

Since parboiling affected Zn TR in milled rice, the potential contribution of biofortified and non-biofortified rice to Zn intake in populations where rice is the main staple food was estimated (Table 4). An estimated per capita daily rice consumption of 420 g for women and 134 g for 2**–**4 years old children from Bangladesh was used as example (Arsenault et al., 2010). Zinc daily requirement was considered as 1390 µg for children aged 4–6 years and 2900 µg for women of child-bearing age (EFSA, 2014). Zinc concentrations of BF and NBF rice used for this estimation was based on the expected Zn level of biofortified rice with full target level of Zn (28 µg.g^−1^ after milling) and a baseline of 16 µg.g^−1^ for milled non-parboiled rice. Average Zn retention used for BF and NBF entries was based on the TR of parboiled and non-parboiled rice at each DOM. For children aged 4–6 years old, biofortified rice could contribute up to 55–61% of the Zn requirement when consuming non-parboiled rice. However, when rice is parboiled, the contribution of biofortified rice to the EAR could be only 38–43% (Table 4). Contribution of biofortified Zn rice to Zn EAR could be up to 70% if consumed as non-parboiled brown rice and only 38% if consumed as parboiled and polished at high levels, which typically occurs in medium and large-scale milling facilities. In contrast, the contribution of non-biofortified extensively milled rice to Zn EAR could be only 22%. For women of child-bearing age, the contribution of BF rice could be 83–91% when non-parboiled and 57–65% when parboiled (Table 4).

**Table 4:** Potential contribution of biofortified (BF) and non-biofortified (NBF) rice to Zn estimated average requirement (EAR) when processed as parboiled and non-parboiled and milled at different degrees of milling (DOM).

Process	Rice type	DOM (%)	Zn retention (%)	Zn content (µg.g^−1^) ^¥^	Daily Zn intake for children (mg)	EAR for children (%)^§^	Daily Zn intake for women (mg)	EAR for women (%)^§^
Brown	BF	0	100	32.4	3.9	70	12.2	106
	NBF	0	100	20.9	2.5	45	7.9	68
Non-parboiled	BF	7.5	87	28.0	3.4	61	10.6	91
	NBF	7.5	77	16.0	1.9	35	6.0	52
	BF	10	79	25.5	3.1	55	9.6	83
	NBF	10	70	14.6	1.8	32	5.5	47
Parboiled	BF	7.5	61	19.8	2.4	43	7.5	65
	NBF	7.5	59	12.2	1.5	27	4.6	40
	BF	10	54	17.4	2.1	38	6.6	57
	NBF	10	49	10.3	1.2	22	3.9	34

^¥^Based in a breading target of 28 µg.g^−1^ for milled BF rice and a baseline of 16 µg.g^−1^ for milled NBF rice. §EFSA estimated average physiological requirement = 2900 µg for women and 1390 µg for children 4–6 yr. Zn in target BF brown rice = 31 µg.g^−1^ and Zn in NBF brown rice = 21 µg.g^−1^. Zn bioavailability = 25%, cooking Zn retention = 90%, rice intake for children = 134 g and rice intake for women = 420 g.

Hossain et al. (2012) reported that farmers in Bangladesh typically under-milled rice that is consumed at the household level (rice which is not sold) whereas rice to be commercialized is typically over-milled to obtain a whiter and longer grain that has a better market price. In regions where this is proven true, biofortified rice consumed by rice producers could have a higher contribution to the Zn EAR% than biofortified rice consumed by net purchasers of rice, as suggested by the results of this study. Considering the large differences in TR due to parboiling and milling methods, potential contribution of biofortified to Zn EAR% should be calculated based on whether the rice is consumed as parboiled or non-parboiled and on the expected DOM in each country or region.

## 4 Conclusion

When rice was parboiled, Zn from the inner endosperm moved towards the outer layers, resulting in lower Zn concentration in milled rice (7.5% and 10% DOM), however Zn TR of biofortified rice remained higher than non-biofortified rice. In general, Fe and PA concentration in milled rice were not affected by parboiling. Based on these findings, it is recommended that rice not be over-milled to ensure a higher intake of Zn, especially if parboiled. Despite some known nutritional benefits of parboiled rice, such as higher vitamin B content, for populations with high Zn deficiency it is prudent to promote the consumption of milled non-parboiled over milled parboiled rice as the latter losses more Zn during milling. Given that biofortified rice could significantly increase the Zn EAR for children and women when consumed either as parboiled or non-parboiled, the production of rice varieties with higher target level for Zn biofortification should be considered for populations that consume mostly parboiled or highly polished rice.

## CRediT authorship contribution statement

**Víctor Taleon:** Conceptualization, Methodology, Formal analysis, Resources, Data curation, Writing - original draft, Project administration. **Sonia Gallego:** Methodology, Validation, Resources, Investigation, Data curation, Writing - review & editing. **Juan Camilo Orozco:** Validation, Investigation, Writing - review & editing. **Cecile Grenier:** Resources, Writing - review & editing, Supervision.

## Declaration of Competing Interest

The authors declare that they have no known competing financial interests or personal relationships that could have appeared to influence the work reported in this paper.
