# Supplementary material for: Retention of Zn, Fe and phytic acid in parboiled biofortified and non-biofortified rice
Source: Food Chem X. 2020 Sep 29;8:100105. doi: 10.1016/j.fochx.2020.100105 (PMC7548297; doi:10.1016/j.fochx.2020.100105)
Supplement: Supplementary data 1 [file mmc1.docx]

Milled non-parboiled rice at 7.5 and 10% DOM

(NPBDOM7.5, NPBDOM10), n = 60

Milled parboiled rice

at 7.5 and 10% DOM (PB13DOM7.5, PB13DOM10), n = 60

Paddy rice (RAW), 2400 g

n = 5 entries x 2 locations x 3 repetitions = 30 RAW batches

RAW soaked to 33% MC

Soaking 2000 g of RAW at 20ºC, 40 h

Parboiled paddy (PB13)

Steaming for 13 min, slow cooling, drying to 11–13% MC

Parboiled paddy (PB16)

Brown parboiled rice (PB13DOM0), n =30

Brown parboiled rice (PB16DOM0), n =30

Dehulling

Dehulling

Brown rice

(NPBDOM0), n = 30

Milled parboiled rice

at 7.5 % DOM (PB16DOM7.5)

n = 30

Steaming for 16 min, fast cooling, drying to 11–13% MC

Dehulling 400 g of RAW

29 g each milling batch, (3–6 milling batches), composited for each target DOM +/- 0.5%

**Supplementary Figure 1.** Summary of the parboiling process for each of the 30 rice samples (five entries, two locations, three processing repetitions) and milling at different degrees of milling (DOM).
