# Supplementary material for: Retention of Zn, Fe and phytic acid in parboiled biofortified and non-biofortified rice
Source: Food Chem X. 2020 Sep 29;8:100105. doi: 10.1016/j.fochx.2020.100105 (PMC7548297; doi:10.1016/j.fochx.2020.100105)
Supplement: Supplementary data 2 [file mmc2.docx]

**Supplementary Figure 2.** Correlation between Zn and Fe concentration in brown rice grain grown in Palmira (A) and Santa Rosa (B), Colombia.
