# Supplementary material for: Retention of Zn, Fe and phytic acid in parboiled biofortified and non-biofortified rice
Source: Food Chem X. 2020 Sep 29;8:100105. doi: 10.1016/j.fochx.2020.100105 (PMC7548297; doi:10.1016/j.fochx.2020.100105)
Supplement: Supplementary data 3 [file mmc3.docx]

**Supplementary Figure 3.** Correlation between Zn concentration in rice (brown, milled non-parboiled and milled parboiled) measured by energy-dispersive X-ray fluorescence spectrometer (XRF) and inductively coupled plasma optical emission spectrometer (ICP-OES).
