# Supplementary material for: Retention of Zn, Fe and phytic acid in parboiled biofortified and non-biofortified rice
Source: Food Chem X. 2020 Sep 29;8:100105. doi: 10.1016/j.fochx.2020.100105 (PMC7548297; doi:10.1016/j.fochx.2020.100105)
Supplement: Supplementary data 4 [file mmc4.docx]

**Supplementary Table 1**

Physical characterization and fat content of whole grain and milled grain of three biofortified and two non-biofortified rice entries grown at two locations in Colombia^¥^.

| **Location** | **Grain Type** | **Grain source code** | **Length**  **(mm)** | **Breadth**  **(mm)** | **L/B**  **ratio** | **Fat in NPBDOM0 (%)** | **Fat in PB13DOM7.5 (%)** | **Whiteness in PB13DOM7.5** |
| --- | --- | --- | --- | --- | --- | --- | --- | --- |
| Palmira | Biofortified | BF1P | 5.6 | 2.5 | 2.2 | 3.3 | 0.64 | 25.4 |
|  | Biofortified | BF2P | 7.3 | 2.4 | 3.1 | 3.2 | 0.84 | 24.0 |
|  | Biofortified | BF3P | 7.4 | 2.5 | 2.9 | 3.6 | 0.82 | 25.5 |
|  | Non-biofortified | NBF1P | 7.1 | 2.2 | 3.2 | 2.7 | 0.84 | 23.4 |
|  | Non-biofortified | NBF2P | 6.7 | 2.3 | 2.8 | 2.2 | 0.80 | 21.9 |
|  | Average Palmira |  | 6.8 | 2.4 | 2.8 | 3.0 | 0.79 | 24.0 |
| Santa Rosa | Biofortified | BF1SR | 5.6 | 2.7 | 2.1 | 3.0 | 0.82 | 29.8 |
|  | Biofortified | BF2SR | 6.7 | 2.3 | 2.9 | 3.1 | 1.02 | 25.4 |
|  | Biofortified | BF3SR | 7.2 | 2.4 | 3.0 | 2.3 | 1.28 | 31.0 |
|  | Non-biofortified | NBF1SR | 7.0 | 2.2 | 3.2 | 2.6 | 0.76 | 27.5 |
|  | Non-biofortified | NBF2SR | 6.9 | 2.2 | 3.1 | 3.4 | 0.84 | 25.6 |
|  | Average Santa Rosa |  | 6.7 | 2.4 | 2.8 | 2.9 | 0.94 | 27.9 |
|  | Average BF |  | 6.6 | 2.5 | 2.7 | 3.1 | 0.90 | 26.9 |
|  | Average NBF |  | 6.9 | 2.2 | 3.1 | 2.7 | 0.81 | 24.6 |

^¥^PB13DOM0 /B= length to breadth ratio of brown rice. BF= biofortified, NBF = non-biofortified. NPBDOM0 = brown non-parboiled rice. PB13DOM7.5 = parboiled rice milled at 7.5% degrees of milling.
