# Supplementary material for: Retention of Zn, Fe and phytic acid in parboiled biofortified and non-biofortified rice
Source: Food Chem X. 2020 Sep 29;8:100105. doi: 10.1016/j.fochx.2020.100105 (PMC7548297; doi:10.1016/j.fochx.2020.100105)
Supplement: Supplementary data 5 [file mmc5.docx]

**Supplementary Table 2**

Phytic acid (PA) in grain of brown parboiled (PB13DOM0 and PB16DOM0), milled non-parboiled (NPBDOM7.5 and NPBDOM10), parboiled milled at 7.5% degrees of milling (PB13DOM7.5, PB16DOM7.5) and parboiled milled at 10% degrees of milling (PB13DOM10) of three biofortified and two non-biofortified rice entries grown at two locations in Colombia^¥^.

| **Location** | **Grain source**  **code** | **PB13DOM0** | **PB16DOM0** | **NPBDOM7.5** | **PB13DOM7.5** | **PB16DOM7.5** | **NPBDOM10** | **PB13DOM10** |
| --- | --- | --- | --- | --- | --- | --- | --- | --- |
|  |  | **PA (mg.g^-1)^** | **PA (mg.g^-1)^** | **PA (mg.g^-1)^** | **PA (mg.g^-1)^** | **PA (mg.g^-1)^** | **PA (mg.g^-1)^** | **PA (mg.g^-1)^** |
| Palmira | BF1P | 10.2 ± 0.8^ab^ | 10.2 ± 0.5^ab^ | 3.1 ± 0.1^c^ | 3.3 ± 0.1^ab^ | 3.4 ± 0.4^b^ | 2.3 ± 0.1^b^ | 2.5 ± 0.2^b^ |
|  | BF2P | 9.8 ± 0.5^ab^ | 10.4 ± 0.1^a^ | 3.0 ± 0.1^c^ | 2.5 ± 0.2^bc^ | 2.8 ± 0.2^bc^ | 1.8 ± 0.1^cde^ | 1.8 ± 0.1^cd^ |
|  | BF3P | 10.5 ± 0.7^a^ | 10.7 ± 0.2^a^ | 4.3 ± 0.0^a^ | 3.7 ± 0.6^a^ | 4.1 ± 0.1^a^ | 2.7 ± 0.0^a^ | 3.0 ± 0.1^a^ |
|  | NBF1P | 8.6 ± 0.7^bcd^ | 9.2 ± 0.3^b^ | 1.8 ± 0.1^g^ | 2.5 ± 0.3^bc^ | 2.4 ± 0.2^cd^ | 1.0 ± 0.1^f^ | 2.0 ± 0.2^c^ |
|  | NBF2P | 9.4 ± 0.8^abc^ | 9.8 ± 0.7^ab^ | 2.5 ± 0.2^ef^ | 2.5 ± 0.4^bc^ | 2.8 ± 0.3^bc^ | 1.7 ± 0.0^de^ | 1.8 ± 0.0^cd^ |
|  | Average Palmira | 9.7 ± 0.7^A^ | 10.1 ± 0.6^A^ | 2.9 ± 0.9^A^ | 2.9 ± 0.6^A^ | 3.1 ± 0.7^A^ | 1.9 ± 0.7 ^A^ | 2.2 ± 0.5^A^ |
| Santa Rosa | BF1SR | 6.2 ± 0.4^e^ | 6.2 ± 0.3^d^ | 2.4 ± 0.1^f^ | 1.6 ± 0.0^d^ | 1.7 ± 0.1^e^ | 2.1 ± 0.1^bc^ | 1.6 ± 0.1^d^ |
|  | BF2SR | 7.6 ± 0.1^cde^ | 8.0 ± 0.1^c^ | 3.2 ± 0.1^c^ | 2.4 ± 0.2^cd^ | 2.6 ± 0.3^cd^ | 1.9 ± 0.2^cd^ | 1.6 ± 0.1^d^ |
|  | BF3SR | 7.0 ± 0.4^de^ | 7.0 ± 0.4^cd^ | 3.6 ± 0.1^b^ | 2.1 ± 0.2^cd^ | 2.2 ± 0.2^cde^ | 2.3 ± 0.1^b^ | 1.6 ± 0.1^d^ |
|  | NBF1SR | 7.1 ± 0.2^de^ | 7.1 ± 0.3^cd^ | 2.9 ± 0.1^cd^ | 1.9 ± 0.2^cd^ | 1.9 ± 0.1^de^ | 1.9 ±0.0^cd^ | 1.5 ± 0.1^d^ |
|  | NBF2SR | 6.7 ± 0.5^e^ | 7.4 ± 0.2^c^ | 2.7 ± 0.0^de^ | 2.0 ± 0.3^cd^ | 2.6 ± 0.3^cd^ | 1.6 ± 0.1^e^ | 1.5 ± 0.0^d^ |
|  | Average Santa Rosa | 6.9 ± 0.5^B^ | 7.1 ± 0.7^B^ | 3.0 ± 0.5^A^ | 2.0 ± 0.3^A^ | 2.2 ± 0.4^A^ | 1.9 ± 0.3^A^ | 1.6 ± 0.1^A^ |
|  | Average BF | 8.5 ± 1.8^A^ | 8.7 ± 2.0^A^ | 3.2 ± 0.6^A^ | 2.6 ± 0.8^A^ | 2.8 ± 0.9^A^ | 2.2 ± 0.3^A^ | 2.0 ± 0.6^A^ |
|  | Average NBF | 8.0 ± 1.3^A^ | 8.4 ± 1.3^A^ | 2.5 ± 0.5^A^ | 2.2 ± 0.3^A^ | 2.4 ± 0.4^A^ | 1.5 ± 0.4^A^ | 1.7 ± 0.2^A^ |

^¥^PB13DOM0 and PB16DOM0 = brown parboiled rice, NPBDOM7.5 = non-parboiled rice at 7.5% degree of milling, PB13DOM7.5 and PB16DOM7.5 = parboiled rice milled at 7.5% degree of milling, NPBDOM10 = non-parboiled rice at 10.0% degree of milling and PB13DOM10 = parboiled rice at 10.0% degree of milling. Different lowercase letters within each column indicate significant differences between entries (*p* < 0.05). Different uppercase letters within each column indicate significant differences between locations and between rice type (*p* < 0.05).
