# Supplementary material for: Retention of Zn, Fe and phytic acid in parboiled biofortified and non-biofortified rice
Source: Food Chem X. 2020 Sep 29;8:100105. doi: 10.1016/j.fochx.2020.100105 (PMC7548297; doi:10.1016/j.fochx.2020.100105)
Supplement: Supplementary data 6 [file mmc6.docx]

**Supplementary Table 3**

Phytic acid (PA) and Zn ratio in grain of brown parboiled (PB13DOM0 and PB16DOM0), milled non-parboiled (NPBDOM7.5 and NPBDOM10), parboiled milled at 7.5% degrees of milling (PB13DOM7.5, PB16DOM7.5) and 10% degrees of milling (PB13DOM10) of three biofortified and two non-biofortified rice entries grown at two locations in Colombia^¥^.

| **Location** | **Grain source**  **code** | **PB13DOM0** | **PB16DOM0** | **NPBDOM7.5** | **PB13DOM7.5** | **PB16DOM7.5** | **NPBDOM10** | **PB13DOM10** |
| --- | --- | --- | --- | --- | --- | --- | --- | --- |
|  |  | **PA:Zn ratio** | **PA:Zn ratio** | **PA:Zn ratio** | **PA:Zn ratio** | **PA:Zn ratio** | **PA:Zn ratio** | **PA:Zn ratio** |
| Palmira | BF1P | 55.6 ± 7.4^a^ | 58.8 ± 4.3^ab^ | 21.4 ± 0.5^ab^ | 28.3 ± 1.5^a^ | 29.5 ± 3.4^a^ | 18.9 ± 1.0^a^ | 24.0 ± 3.4^a^ |
|  | BF2P | 47.8 ± 6.8^a^ | 52.5 ± 3.6^b^ | 18.8 ± 1.1^b^ | 16.3 ± 1.5^b^ | 18.4 ± 3.3^b^ | 11.2 ± 0.5^c^ | 14.0 ± 0.6^b^ |
|  | BF3P | 52.0 ± 8.2^a^ | 48.2 ± 2.4^b^ | 24.2 ± 1.9^a^ | 26.8 ± 3.0^a^ | 27.9 ± 1.7^a^ | 17.5 ± 0.8^ab^ | 22.7 ± 1.5^a^ |
|  | NBF1P | 62.6 ± 4.7^a^ | 67.9 ± 7.2^a^ | 18.9 ± 0.8^b^ | 31.0 ± 3.9^a^ | 30.2 ± 3.0^a^ | 12.0 ± 0.7^c^ | 26.1 ± 3.5^a^ |
|  | NBF2P | 64.8 ±9.9^a^ | 70.1 ± 7.9^a^ | 21.4 ± 3.2^ab^ | 26.5 ± 2.2^a^ | 33.5 ± 3.2^a^ | 14.9 ± 2.2^b^ | 21.5 ± 1.8^a^ |
|  | Average Palmira | 56.6 ± 7.1^A^ | 59.5 ± 9.5^A^ | 20.9 ±2.2^A^ | 25.8 ± 5.6^A^ | 27.9 ± 5.7^A^ | 14.9 ± 3.4^A^ | 21.7 ± 4.6^A^ |
| Santa Rosa | BF1SR | 18.3 ± 1.2^b^ | 18.8 ± 1.2^c^ | 8.1 ± 0.2^e^ | 8.3 ± 1.3^c^ | 8.6 ± 1.4^c^ | 7.3 ± 0.5^de^ | 8.4 ± 0.5^c^ |
|  | BF2SR | 21.9 ± 1.4^b^ | 22.3 ± 1.4^c^ | 9.3 ± 1.2^de^ | 10.2 ± 0.5^c^ | 10.4 ± 2.4^c^ | 6.4 ± 0.6^e^ | 7.8 ± 0.7^c^ |
|  | BF3SR | 19.1 ± 1.0^b^ | 18.8 ± 0.5^c^ | 10.5 ± 1.1^cde^ | 9.6 ± 0.7^c^ | 9.6 ± 1.0^c^ | 7.5 ± 1.1^de^ | 7.6 ± 0.6^c^ |
|  | NBF1SR | 28.7 ± 1.2^b^ | 28.6 ± 0.8^c^ | 14.0 ± 1.7^c^ | 13.9 ± 1.5^bc^ | 13.0 ± 0.2^bc^ | 9.7 ± 0.3^cd^ | 11.4 ± 0.8^bc^ |
|  | NBF2SR | 26.2 ± 3.3^b^ | 28.7 ± 1.9^c^ | 12.7 ± 0.4^cd^ | 12.2 ± 2.0^bc^ | 13.2 ± 3.1^bc^ | 7.8 ± 0.7^de^ | 10.1 ± 0.6^bc^ |
|  | Average Santa Rosa | 22.9 ± 4.5^B^ | 23.4 ± 5.0^B^ | 10.9 ± 2.4^B^ | 10.9 ± 2.2^B^ | 11.0 ± 2.0^B^ | 7.7 ± 1.2^B^ | 9.1 ± 1.7^B^ |
|  | Average BF | 35.8 ± 17.7^A^ | 36.6 ± 18.5^A^ | 15.4 ±6.9^A^ | 16.6 ± 8.9 ^A^ | 17.4 ± 9.4^A^ | 11.5 ± 5.5^A^ | 14.1 ± 7.6^A^ |
|  | Average NBF | 45.6 ± 21.0^A^ | 48.8 ± 23.3^A^ | 16.8 ±4.1^A^ | 20.9 ± 9.3^A^ | 22.5 ± 10.9^A^ | 11.1 ± 3.1^A^ | 17.3 ± 7.8^A^ |

^¥^PB13DOM0 and PB16DOM0 = brown parboiled rice, NPBDOM7.5 = non-parboiled rice at 7.5% degree of milling, PB13DOM7.5 and PB16DOM7.5 = parboiled rice milled at 7.5% degree of milling, NPBDOM10 = non-parboiled rice at 10.0% degree of milling and PB13DOM10 = parboiled rice at 10.0% degree of milling. Different lowercase letters within each column indicate significant differences between entries (*p* < 0.05). Different uppercase letters within each column indicate significant differences between locations and between rice type (*p* < 0.05).
