# Supplementary material for: Retention of Zn, Fe and phytic acid in parboiled biofortified and non-biofortified rice
Source: Food Chem X. 2020 Sep 29;8:100105. doi: 10.1016/j.fochx.2020.100105 (PMC7548297; doi:10.1016/j.fochx.2020.100105)
Supplement: Supplementary data 7 [file mmc7.docx]

**Supplementary Table 4**

Phytic acid (PA) and Fe ratio in grain of parboiled brown (PB13DOM0 and PB16DOM0), non-parboiled milled (NPBDOM7.5 and NPBDOM10), and parboiled milled at 7.5% degrees of milling (PB13DOM7.5, PB16DOM7.5) and 10% degrees of milling (PB13DOM10) of three biofortified and two non-biofortified rice entries grown at two locations in Colombia^¥^.

| **Location** | **Grain source**  **code** | **PB13DOM0** | **PB16DOM0** | **NPBDOM7.5** | **PB13DOM7.5** | **PB16DOM7.5** | **NPBDOM10** | **PB13DOM10** |
| --- | --- | --- | --- | --- | --- | --- | --- | --- |
|  |  | **PA:Fe ratio** | **PA:Fe ratio** | **PA:Fe ratio** | **PA:Fe ratio** | **PA:Fe ratio** | **PA:Fe ratio** | **PA:Fe ratio** |
| Palmira | BF1P | 83.1 ± 7.7^a^ | 85.2 ± 7.8^a^ | 56.4 ± 12.3^abc^ | 69.8 ± 6.7^a^ | 90.1 ± 9.9^a^ | 67.9 ± 5.3^a^ | 82.0 ± 14.5^bcd^ |
|  | BF2P | 82.3 ± 8.7^a^ | 86.7 ± 0.8^a^ | 70.6 ± 3.8^ab^ | 62.0 ± 4.5^ab^ | 74.3 ± 12.5^abc^ | 64.9 ± 7.2^a^ | 68.4 ± 7.0^cde^ |
|  | BF3P | 74.4 ± 5.2^ab^ | 72.4 ± 1.9^ab^ | 70.9 ± 14.1^ab^ | 70.5 ± 11.2^a^ | 84.3 ± 27.5^ab^ | 69.7 ± 27.2^a^ | 113.7 ± 4.7^a^ |
|  | NBF1P | 80.1 ± 7.0^a^ | 85.6 ± 11.0^a^ | 72.2 ± 8.2^ab^ | 80.4 ± 23.5^a^ | 85.9 ± 12.1^ab^ | 58.4 ± 9.8^a^ | 104.4 ± 19.0^ab^ |
|  | NBF2P | 80.8 ± 8.7^a^ | 87.2 ± 4.2^a^ | 75.9 ± 3.9^a^ | 71.1 ± 12.2^a^ | 93.7 ± 5.9^a^ | 53.4 ± 31.8^a^ | 93.3 ± 6.9^abc^ |
|  | Average Palmira | 80.1 ± 3.4^A^ | 83.4 ± 6.2^A^ | 69.2 ± 7.4^A^ | 70.7 ± 6.5 ^A^ | 85.7 ± 7.3^A^ | 62.8 ± 6.8 ^A^ | 92.4 ± 17.9^A^ |
| Santa Rosa | BF1SR | 46.9 ± 6.9^c^ | 50.7 ± 3.7^c^ | 42.5 ± 4.9^c^ | 32.0 ± 2.5^b^ | 41.6 ± 2.0^c^ | 46.8 ± 4.4^a^ | 40.4 ± 1.9^e^ |
|  | BF2SR | 54.7 ± 8.0^bc^ | 57.6 ± 2.3^bc^ | 51.1 ± 7.7^bc^ | 47.1 ± 5.5^ab^ | 54.1 ± 7.5^bc^ | 51.3 ± 5.0^a^ | 45.9 ± 2.2^e^ |
|  | BF3SR | 47.7 ± 5.2^c^ | 46.7 ± 3.6^c^ | 53.9 ± 5.3^bc^ | 46.7 ± 7.9^ab^ | 51.0 ± 7.5^bc^ | 61.5 ± 9.7^a^ | 62.0 ± 8.9^de^ |
|  | NBF1SR | 74.9 ± 2.6^ab^ | 76.2 ± 4.0^a^ | 77.9 ± 3.1^a^ | 66.3 ± 15.6^ab^ | 63.4 ± 2.2^abc^ | 74.3 ± 3.0^a^ | 67.4 ± 15.1^cde^ |
|  | NBF2SR | 72.6 ± 7.5^ab^ | 82.8 ± 6.2^a^ | 77.4 ± 1.5^a^ | 60.8 ± 16.0^ab^ | 76.8 ± 12.7^ab^ | 81.8 ± 5.6^a^ | 75.2 ± 4.3^bcd^ |
|  | Average Santa Rosa | 59.4 ± 13.5^B^ | 62.8 ± 15.9^B^ | 60.6 ± 16.1^B^ | 50.6 ± 13.4^B^ | 57.4 ± 13.4^B^ | 63.1 ± 14.8 ^A^ | 58.2 ± 14.6^B^ |
|  | Average BF | 64.8 ± 17.0^B^ | 66.6 ± 17.4^B^ | 57.6 ± 11.2^B^ | 54.7 ± 15.3^A^ | 65.9 ± 19.7^A^ | 60.4 ± 9.3^A^ | 68.7 ± 26.7^A^ |
|  | Average NBF | 77.1 ± 4.0^A^ | 82.9 ± 4.8^A^ | 75.8 ± 2.6^A^ | 69.6 ± 8.3^A^ | 79.9 ± 13.0^A^ | 67.0 ± 13.3^A^ | 85.1 ± 16.9^A^ |

^¥^PB13DOM0 and PB16DOM0 = brown parboiled rice, NPBDOM7.5 = non-parboiled rice at 7.5% degree of milling, PB13DOM7.5 and PB16DOM7.5 = parboiled rice milled at 7.5% degree of milling, NPBDOM10 = non-parboiled rice at 10.0% degree of milling and PB13DOM10 = parboiled rice at 10.0% degree of milling. Different lowercase letters within each column indicate significant differences between entries (*p* < 0.05). Different uppercase letters within each column indicate significant differences between locations and between rice type (*p* < 0.05).
